# A three-gene signature reveals changes in the tumor immune microenvironment in the progression from NAFLD to HCC

**DOI:** 10.1038/s41598-023-49358-w

**Published:** 2023-12-15

**Authors:** Lijuan Liu, Haonan Tang, Kui Wang, Jiaying Liu, Ningbin Luo, Guanqiao Jin

**Affiliations:** Guangxi Medical University Cancer Hospital, Nan Ning, Guangxi Zhuang Autonomous Region China

**Keywords:** Cancer therapy, Oncogenes, Tumour biomarkers

## Abstract

Hepatocellular carcinoma (HCC) is one of the most dangerous malignant tumors. The incidence rates of obesity related NAFLD and NASH are increasing year by year, and they are the main risk factors for HCC at present. Finding the mechanism of malignant transformation of NAFLD and NASH is helpful for early prevention and diagnosis. In this study, we performed differential analysis using NAFLD data, NASH data, and HCC data to identify crossover differential genes. Then, using the clinical data of TCGA, a prognostic risk prediction model of three genes (TEAD4, SOCS2, CIT) was constructed, and survival analysis and receiver operating characteristic curves were drawn. The prognostic model was validated using ICGC, GSE116174 and GSE54236 datasets. In addition, we assessed immune status and function in high- and low-risk populations using a prognostic model. Moreover, we assessed the expression of CIT in clinical samples and HCC cell lines and validated its role in HCC development. Our study elucidates the important role of the tumor immune microenvironment in the development of NAFLD/NASH to HCC, deepens the understanding of the pathogenesis of NAFLD/NASH development to HCC, and is helpful for clinical management and decision-making.

## Introduction

Worldwide, hepatocellular carcinoma (HCC) is one of the most common and deadly malignancies. In recent years, there are more and more molecular targets for HCC, but the prognosis of HCC patients has not been greatly improved. The important reason is that many HCC patients are already in the advanced stage when they receive treatment. With the changes in human life and eating habits, the incidence of nonalcoholic fatty liver disease (NAFLD) and nonalcoholic steatohepatitis (NASH) with obesity progression is increasing. Although NAFLD and NASH are benign lesions, they can progress to liver fibrosis and even HCC without intervention^1^. However, two studies in Germany and Japan showed that about 41–49% of HCC patients with NAFLD were without cirrhosis^2,3^ In several other studies, 20–65% of non-cirrhotic NASH patients were diagnosed with HCC^4–6^. This suggests that NAFLD or NASH can directly transform into HCC without going through the cirrhosis stage. Therefore, to ascertain the mechanism of HCC progression and malignant transformation of benign liver lesions is very important for early diagnosis of HCC and saving the prognosis of HCC patients.

It is generally believed that DNA damage, chronic inflammation, autophagy, and oxidative stress caused by persistent immune abnormalities are the key pathogenesis of NAFLD to HCC^7–9^. A mouse study showed that NKT cells and CD8^+^ T cells were activated in High fat diet-fed mice, leading to the occurrence of NASH, and then the activation of NF-κB signaling pathway promoted the transformation of NASH to HCC^10^. Additionally, CD4^+^ T cells have been demonstrated to inhibit the development of HCC and play a role in tumor regression. NASH has also been shown to cause a selective loss of intrahepatic CD4^+^ T cells, and hasten carcinogenesis in liver-specific MYC transgenic mice^11–13^. Therefore, understanding the mechanism of immune alteration from NAFLD to HCC development will lead to better approaches to prevent HCC occurrence and progression.

In this study, we used NAFLD data from GSE89632 and GSE126848, NASH data from GSE17470 and GSE126848, and HCC data from The Cancer Genome Atlas (TCGA) database to find out the crossover differential genes. Then, using the clinical data of TCGA, a prognostic risk prediction model of three genes (TEAD4, SOCS2, CIT) was constructed, and receiver operating characteristic curves (ROC) and survival analysis were drawn. The prognostic model was validated using International Cancer Genome Consortium (ICGC), GSE116174 and GSE54236 datasets. In addition, we assessed immune status and function in the high- and low-risk groups using a prognostic model. Our study clarifies the important role of tumor immune microenvironment (TIME) in the development of NAFLD to HCC, and deepens the understanding of the pathogenesis of NAFLD/NASH to HCC, which can help in clinical early diagnosis and treatment.

## Materials and methods

### Data acquisition

We downloaded the data of HCC patients from TCGA database. Moreover, the NAFLD and NASH datasets were obtained with complete exclusion criteria (alcohol consumption, specific diseases that can cause NAFLD such as viral hepatitis, end-stage liver diseases such as liver failure, those with underlying heart disease, or those with incomplete relevant information) and inclusion criteria (pathological diagnosis). More specifically, we obtained the information of NAFLD patients from GSE89632 and GSE126848, NASH from GSE17470 and GSE126848 for patient information, and information on liver fibrosis grading in NASH patients was obtained from GSE89632. We then selected ICGC (Asia), GSE54236 (Asia), and GSE116174 (Europe) patient sets from different regions (independent of TCGA (USA)) for further model validation.

### Genes associated with prognosis and differentially expressed genes

Differentially expressed gene analysis (|logFC|> 1, adjusted *p* value < 0.05] was performed using the "limma" R package. Model-related prognosis was determined using the 'survival' R package. The prognostic model (risk score = 0.05957332 * TEAD4 expression − 0.233885624 * SOCS2 expression + 0.38039386 * CIT expression) was constructed based on univariate and multivariate Cox regression analysis of TCGA.

### Examining immune cell infiltration and gene enrichment analysis

We analyze gene enrichment in high- and low-risk groups using data from TCGA. Using the Single-Sample Gene Set Enrichment Analysis (ssGSEA) function in the "GSVA" R package. By analyzing the correlation between risk scores and immune-related scores using the "ESTIMATE" R program, additional proof for the potential function of risk scores in HCC TIME was discovered. Immune signatures were examined using the ssGSEA approach, tumor-initiating cell subtypes were assessed using the CIBERSORT method, and 29 genes involved in immune checkpoint blockage were examined.

### Patients and tissue samples

Between January 2021 and January 2022, 40 pairings of HCC sample and corresponding nontumor tissues were acquired with the signed explicit consent of HCC patients at Guangxi Medical University Cancer Hospital (Nanning, China). Preoperative chemotherapy or radiation were not administered to any of the participants in this research. The HCC diagnosis was confirmed by two pathologists. Guangxi Medical University Cancer Hospital's ethical committee granted its approval to the study, which was carried out in accordance with the Helsinki Declaration. These tissue samples were used for subsequent Western blot analysis (WB), RNA extraction and real-time quantitative PCR (qRT‒PCR).

### WB, RNA extraction and qRT‒PCR

For electrophoresis, the material was put to a 10% gel. The protein was electrophoretically separated, then moved to a membrane (Millipore, #IPVH00010) where it was hybridized with the CIT antibody (Proteintech, # 20033-1-AP). Finally, an ECL kit (Thermo, #34096) was used to identify the membrane signal.

After lysing the sample with TRIzol (Thermo, Ambion), total RNA was recovered, and reverse transcription was carried out in accordance with the product's instructions using a cDNA synthesis kit (Thermo, #K1622). Primer sets for CIT Antisense is 5′-TCAGCTATGGTGTCGGAATACT3′ and sense is 5′-ATATGGAGCGCGGAATCCTTT-3′.

### Cell culture

The liver cancer cell line was obtained from the Cell Bank of the Chinese Academy of Sciences (Shanghai, China). The cell line has been tested and validated using short tandem repeat (STR) analysis. The HCC cells were cultured in DMEM/high glucose medium (Hyclone, USA) in a humidified incubator at a temperature of 37 °C and a CO2 concentration of 5%. Before culture, approximately 10% FBS (fetal bovine serum, Gibco USA) and penicillin–streptomycin (100 U/mL and 100 μg/mL respectively) were added to the DMEM/high glucose medium. The cells were counted under a microscope.

### Testing the proliferation of HCC cells

After determining the formation of colonies through regular cultivation, the transfected HCC cells were digested with trypsin, centrifuged, counted, and then replanted at a density of 500 cells per 6 cm dish. After 12 days, the cell colonies (each colony containing at least 50 cells) were fixed with 37% methanol and stained with 0.1% crystal violet. The CCK-8 assay was performed using the CCK8 kit (Dojindo, CK04) in accordance with the manufacturer's instructions.

### Statistical analysis

The software used for statistical analysis was GraphPad Prism 8.0 (GraphPad Software, San Diego, California, USA) and SPSS 24.0 (SPSS Inc., Chicago, Illinois, USA). For comparison of continuous variables, unpaired Student's t-test was used. The “survival” R package is used for Kaplan–Meier or Cox regression analysis. To compare data between two paired or unpaired groups, we utilized Student’s *t* test. Based on two-sided *p* values, a significance threshold of 0.05 was chosen. *p < 0.05; **p < 0.01; ***p < 0.001.

### Ethics approval and consent to participate

This study was approved by the Ethics Committee of Guangxi Medical University Cancer Hospital (permit number:KY 2023470). The tissue samples were obtained with written informed consent from each patient.

## Results

### Screening of prognostic genes related to NAFLD/NASH to HCC

**Figure 1:**
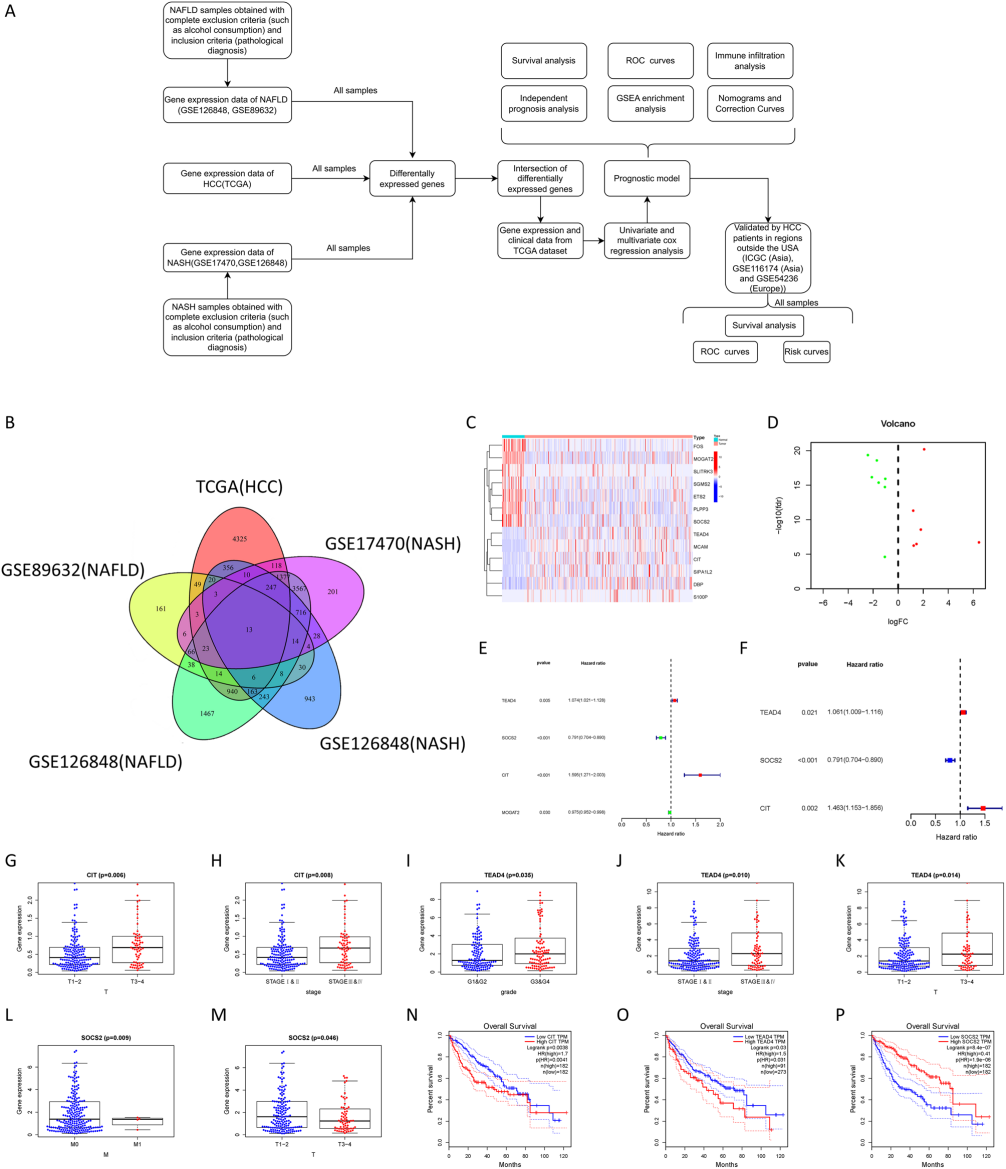
Screening of prognostic genes related to NAFLD/NASH to HCC. (**A**) Flowchart of the study. (**B**) Venn diagram showing differential genes common to NAFLD, NASH, and HCC. (**C**, **D**) Heatmap (using R version 4.2.1: https://www.r-project.org/) and volcano map showing differential genes in TCGA. E–F Univariate and multivariate COX regression analysis in TCGA. G-M Clinical correlation analysis based on gene expression. N-P Survival analysis based on gene expression.

In order to explore the key targets regulating the malignant transformation of benign liver lesions, we tried to find the common differential genes (DEGs) in the progression of benign liver lesions and HCC. We used the "limma" R package to screen 7668 DEGs in HCC from the TCGA database, 458 and 8902 DEGs in NAFLD from GSE89632 and GSE126848, and 2804 and 6396 DEGs in NASH from GSE17470 and GSE126848, respectively (log foldchange > 1, *p* < 0.05) (Fig. 1A). Thirteen DEGs were identified as key genes regulating NAFLD/NASH malignant transformation (Fig. 1B–D). Then we used TCGA HCC data to perform univariate and multivariate COX regression analysis, and identified TEAD4, SOCS2, and CIT as the key genes associated with NAFLD/NASH malignant transformation and HCC prognosis (Fig. 1E, F). Clinical correlation analysis showed that TEAD4 expression was correlated with high T stage and stage and grade of HCC tumors, SOCS2 expression was negatively correlated with lymph node metastasis and T stage of HCC tumors, and CIT was positively correlated with T stage and stage of HCC tumors (Fig. 1G–M). Among them, TEAD4 (HR = 1.074) and CIT (HR = 1.595) are risk factors for the prognosis of HCC, and their high expression leads to poor prognosis of HCC, while SOCS2 (HR = 0.791) is a protective factor, and its high expression improves the prognosis of HCC patients (Fig. 1N–P).

### Construction of a prognostic model

**Figure 2:**
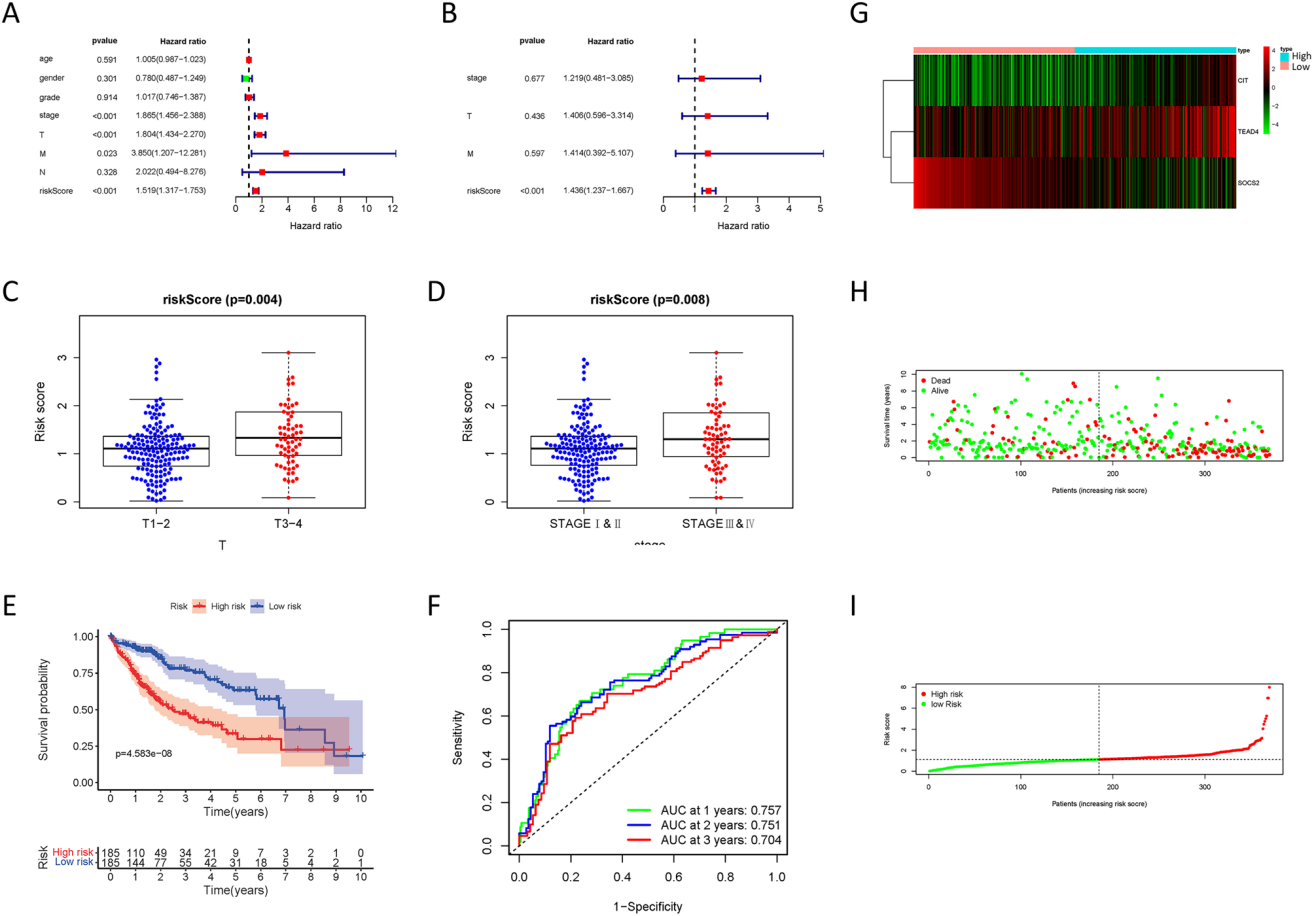
Construction of a prognostic model. (**A**, **B**) Univariate and multivariate COX regression analysis based on clinical factors. (**C**, **D**) Clinical correlation analysis based on risk score. (**E**, **F**) Survival curve and ROC curve based on risk score. (**G**–**I**) Gene expression and survival status based on risk score.

Next, we constructed a HCC risk prognosis model through TEAD4, SOCS2, and CIT, and the risk score = (0.05957332 * TEAD4 expression − 0.233885624 * SOCS2 expression + 0.38039386 * CIT expression). Univariate cox regression analysis using TCGA data showed risk score was a risk factor for HCC (HR = 1.519), and multivariate COX regression analysis showed that risk score was an independent prognostic factor for HCC (HR = 1.436) (Fig. 2A, B). In addition, HCC patients with high risk score showed higher T stage and stage (Fig. 2C, D). Survival analysis showed that HCC patients with high risk score had worse prognosis compared with HCC patients with low risk score (Fig. 2E). The ROC curves of 1, 2, and 3 years (the areas under the ROC curve = 0.757, 0.751, 0.704) showed that our prognostic model was reliable (Fig. 2F). Figure 2G–I shows the expression of TEAD4, SOCS2 and CIT in HCC samples, and the survival status of HCC patients based on risk score.

### Prognostic model verification

**Figure 3:**
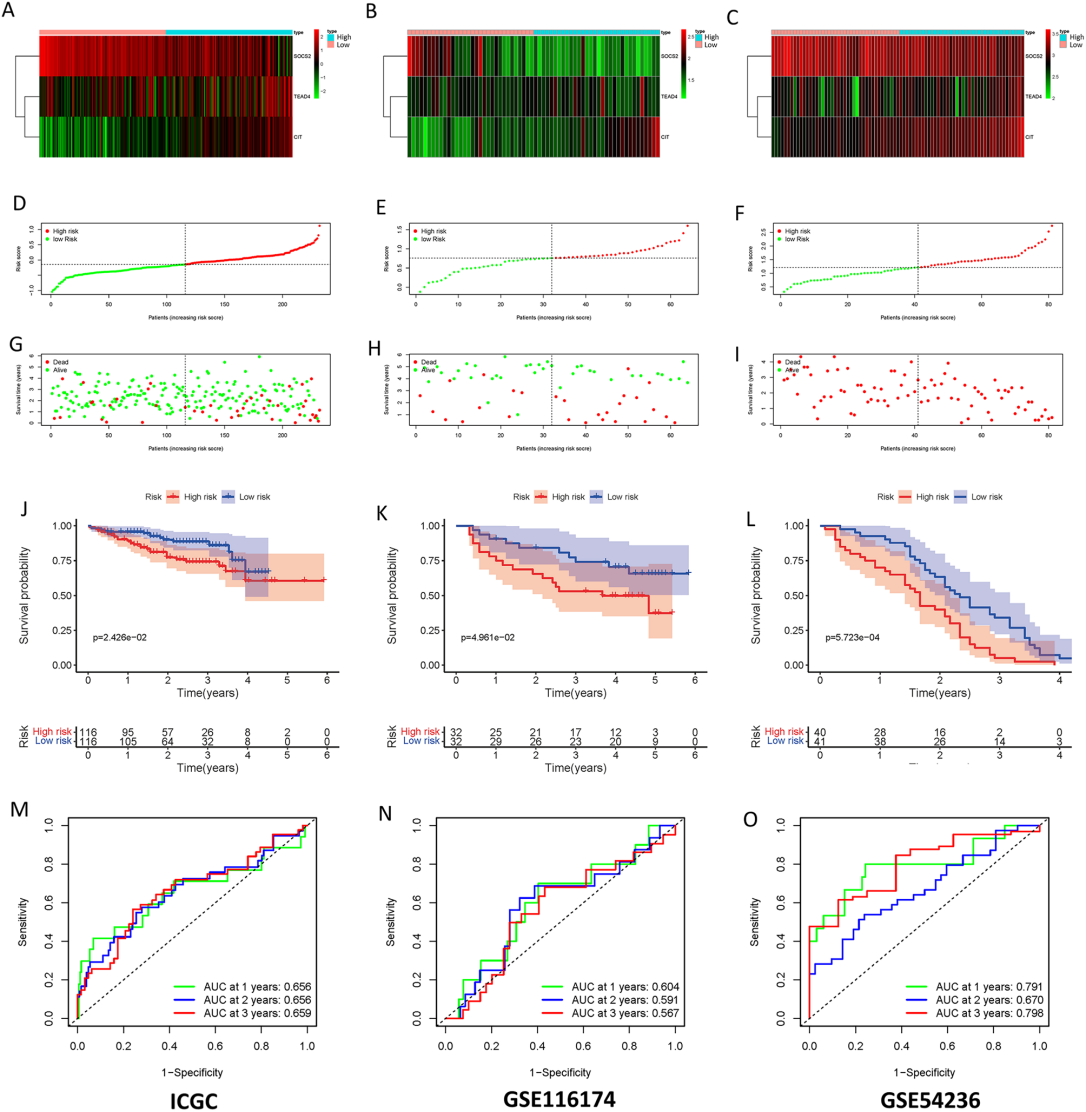
Prognostic model verification. Gene expression heat map (using R version 4.2.1: https://www.r-project.org/) for HCC samples from ICGC (**A**), GSE116174 (**B**), and GSE54236 (**C**). (**D**–**F**) Risk ratings for HCC samples across the three datasets are distributed. (**G**–**I**) Relationship between risk score, HCC sample status, and survival time in the aforementioned three datasets. (**J**–**L**) Samples from the high- and low-risk categories in the aforementioned three datasets' Kaplan–Meier survival curves. (**M**–**O**) The ROC curve of the risk score for samples with HCC.

Then we validated our prognostic model with external datasets (ICGC, GSE116174, GSE54236). Our results showed that higher risk scores were associated with fewer surviving samples and more dead samples (Fig. 3A–I). The prognosis of low-risk samples was much better than that of high-risk groups (Fig. 3J–L). In the ICGC dataset, the areas under the ROC curve for 1, 2, and 3 years are 0.656, 0.656, and 0.659, respectively; in the GSE116174 dataset, they are 0.604, 0.591, and 0.567; in the GSE54236 dataset, they are 0.791, 0.670, and 0.798, respectively (Fig. 3M–O). These data suggest that our model has excellent prognostic value for HCC patients.

### Nomogram construction and validation

**Figure 4:**
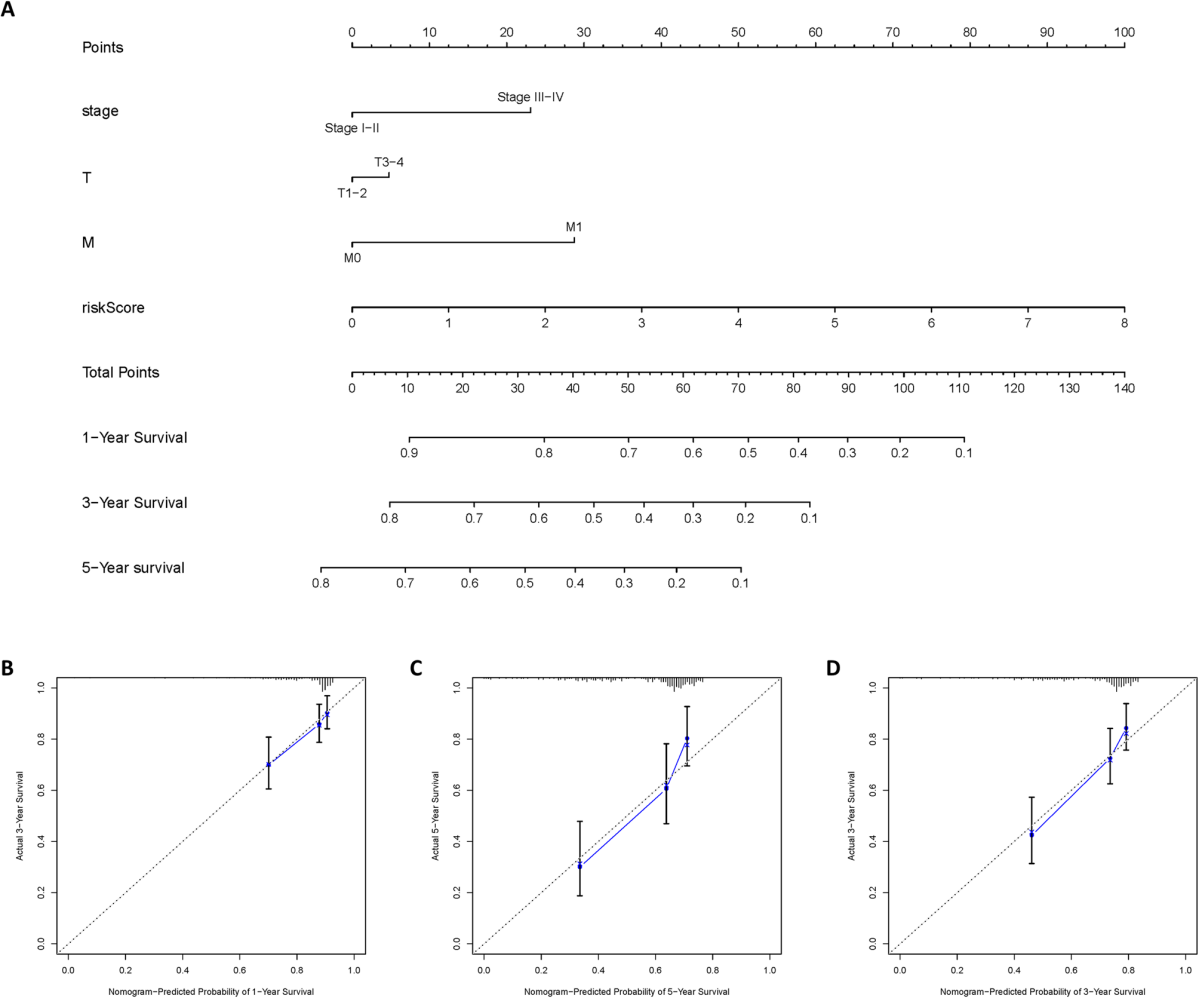
Nomogram construction and validation. (**A**) Nomogram showing survival scores for clinical factors. (**B**–**D**) Correction curves showing the predictive accuracy of 1, 3, and 5-year survival.

For the convenience of clinicians, we quantified the prognostic model and incorporated other independent prognostic factors to develop the nomogram. Nomograms for OS at 1, 3, and 5 years are shown in Fig. 4A. The concordance index (C-index) of the nomogram was 0.7 (se = 0.033), indicating a trustworthy predictive power of the nomogram to a certain extent. In addition, the calibrate curves at 1, 3 and 5 years showed high accuracy (Fig. 4B–D).

### The characteristics of the tumor immunological environment and immune infiltration are highly correlated with risk score

**Figure 5:**
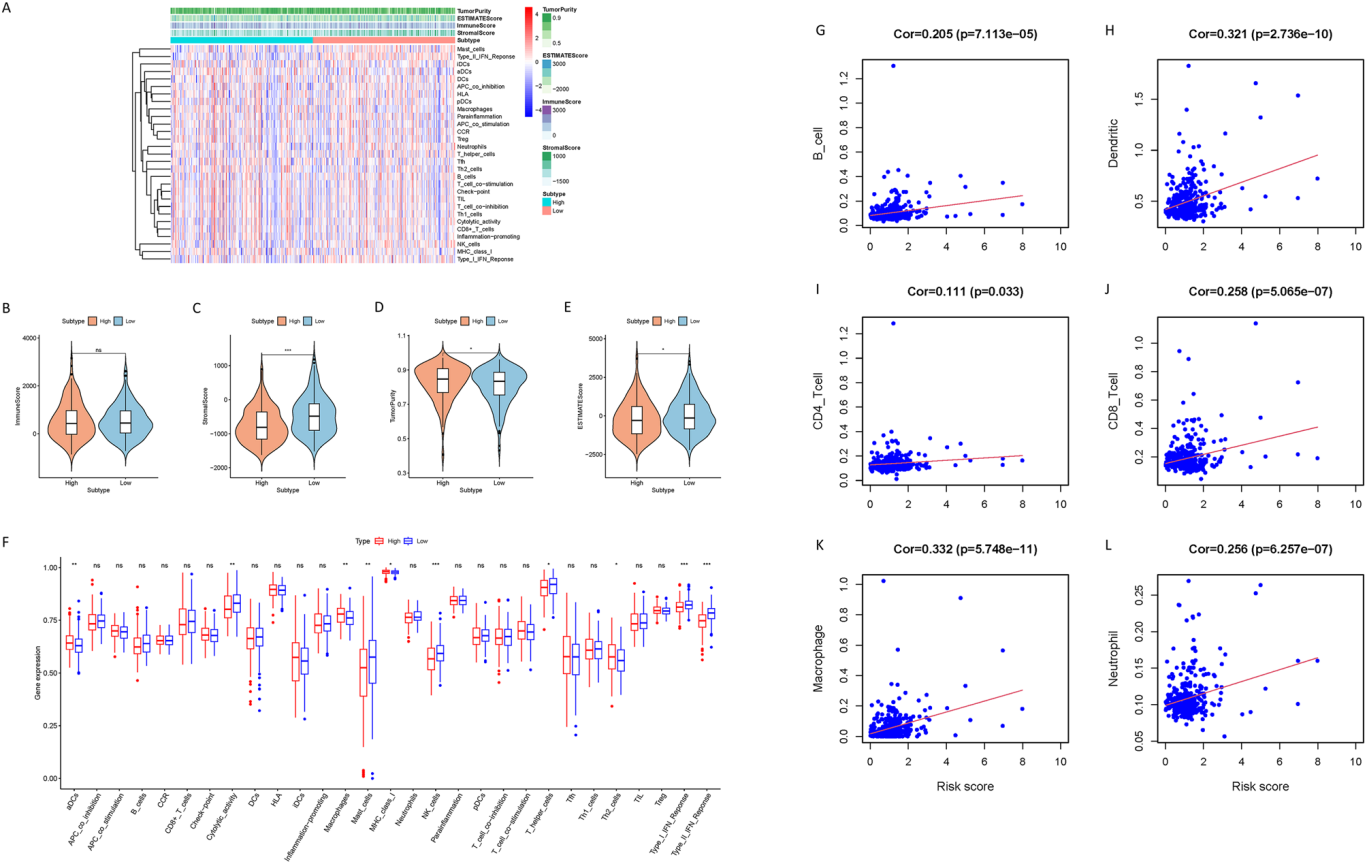
The characteristics of the tumor immunological environment and immune infiltration are highly correlated with risk score. (**A**) Heat map (using R version 4.2.1: https://www.r-project.org/) distribution of risk score and various immune scores. (**B**–**E**) Comparison of estimate score, stromal score, immune score, and tumor purity between high-risk groups and low-risk groups. (**F**) The relationship between immune function, immune cell infiltration and risk score in TCGA cohort. (**G**–**L**) Association between this signature and CD4^+^ T cells, B cells, CD8^+^ T cells, macrophages cells, dendritic cells and neutrophil cells.

Given that immune abnormalities are the key mechanism for the development of NAFLD/NASH to HCC, we performed an immune-related analysis based on risk score for HCC samples. Heatmaps showing the distribution of immune cells, immune function, and immune score in high- and low-risk groups (Fig. 5A). Compared with the low-risk group, the high-risk group had lower ESTIMATEScore, StromalScore, but increased TumorPurity, while ImmuneScore was not significantly different (Fig. 5B–E). Through ssGSEA analysis, the infiltration levels of aDCs, macrophages, and Th2 cells in the high-risk group increased, while the infiltration levels of mast cells, NK cells, and T helper cells decreased. In terms of immune pathways, MCH I was activated in the high-risk group, IFN I and II, and cytolytic activity, were inhibited (Fig. 5F). We discovered through correlation analysis that there was a substantial link between risk markers and infiltrating B cells (r = 0.205), dendritic cells (r = 0.321), CD4 + T cells (r = 0.111), CD8 + T cells (r = 0.258), macrophages (r = 0.332), neutrophils (r = 0.256) (Fig. 5G–L). These findings imply that immunological responses, immune infiltration, and TIME signatures are all substantially correlated with risk scores in HCC.

### Risk score is associated with multiple pathways

**Figure 6:**
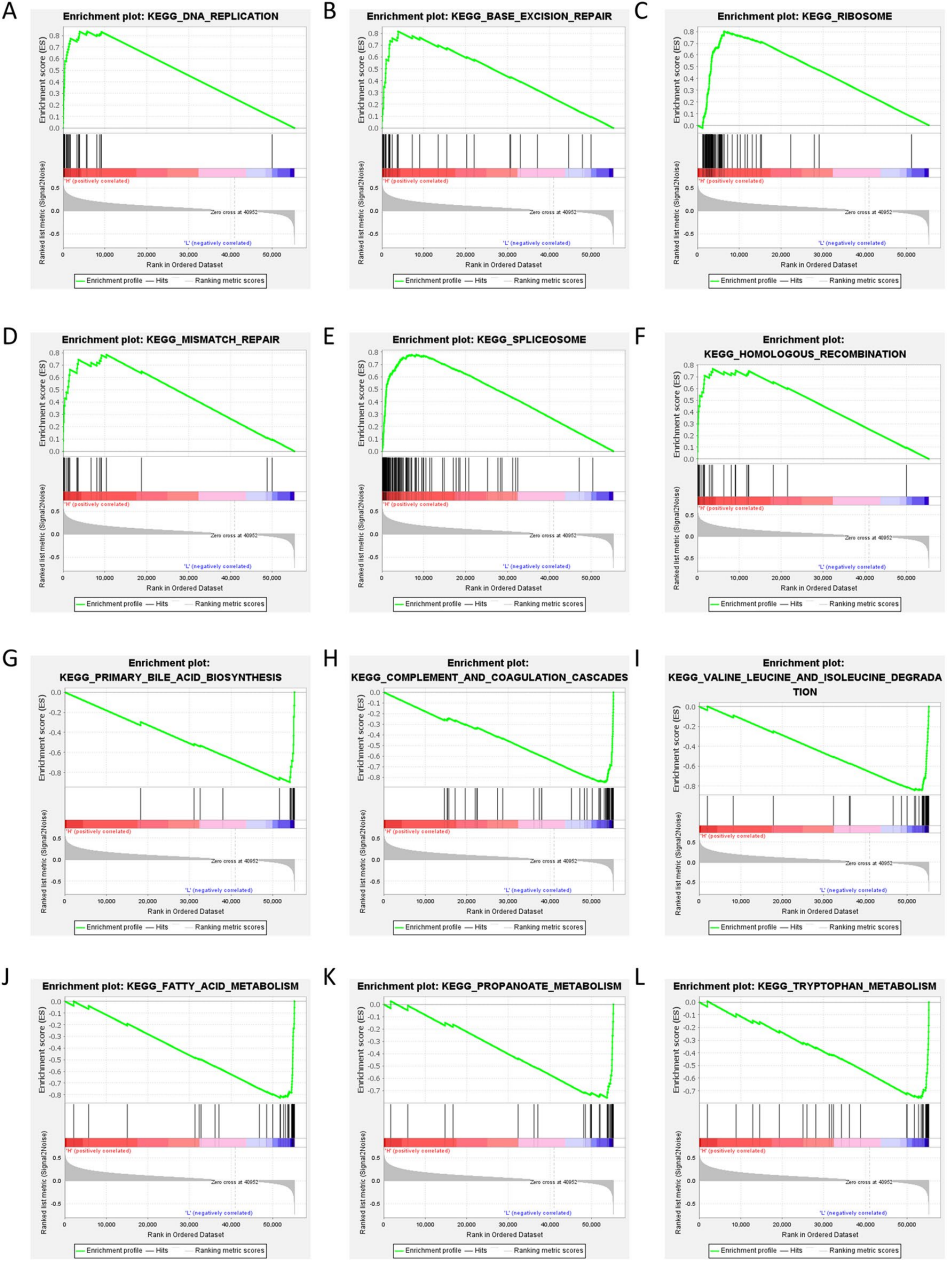
Risk score is associated with multiple pathways. (**A**–**F**) GSEA analysis reveals 6 pathways in KEGG database (www.kegg.jp/kegg/kegg1.html)^31–33^ activated by high-risk group in TCGA. (**G**–**L)** GSEA analysis reveals 6 pathways in KEGG database (www.kegg.jp/kegg/kegg1.html)^31–33^suppressed by high-risk group in TCGA.

In order to find out the deeper mechanism of the poor prognosis of patients in the high-risk group, we conducted GSEA enrichment analysis by risk score, and the results showed that KEGG_DNA_REPLICATION, KEGG_BASE_EXCISION_REPAIR, KEGG_RIBOSOME, KEGG_MISMATCH_REPAIR, KEGG_SPLICEOSOME, KEGG_HOMOLOGOUS_RECOMBINATION and other pathways were significantly activated in patients in the high-risk group (Fig. 6A–F). In the high-risk group, multiple metabolic pathways such as KEGG_PRIMARY_BILE_ACID_BIOSYNTHESIS, KEGG_COMPLEMENT_AND_COAGULATION_CASCADES, KEGG_VALINE_LEUCINE_AND_ISOLEUCINE_DEGRADATION, KEGG_FATTY_ACID_METABOLISM, KEGG_PROPANOATE_METABOLISM, KEGG_TRYPTOPHAN_TA were significantly inhibited (Fig. 6G–L).

### Expression patterns of key genes of malignant transformation of benign liver lesions in NAFLD, NASH and HCC

**Figure 7:**
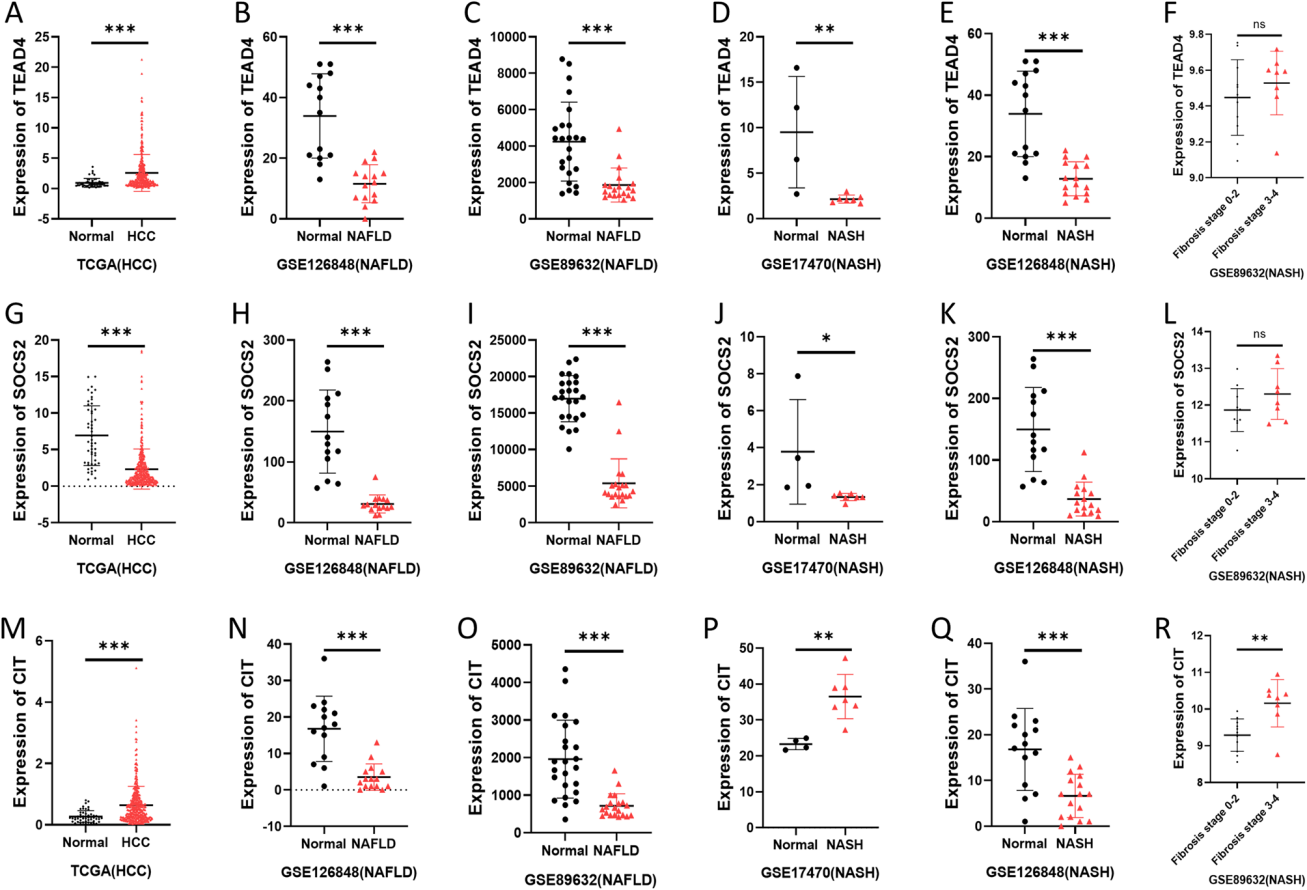
Expression patterns of key genes of malignant transformation of benign liver lesions in NAFLD, NASH and HCC. TEAD4 (**A**–**F**), SOCS2 (**G**–**L**), and CIT (**M**–**R**) gene expression levels in NAFLD, NASH and HCC datasets.

We then analyzed the expression patterns of TEAD4, SOCS2, and CIT in NAFLD, NASH, and HCC. Our results showed that TEAD4 expression was decreased in NAFLD and NASH, but increased in HCC (Fig. 7A–E). The expression of SOCS2 was significantly decreased in NAFLD, NASH and HCC, indicating that the persistent low expression of SOCS2 led to the malignant progression of benign liver lesions (Fig. 7G–K). CIT expression was decreased in two NAFLD datasets (GSE89632 and GSE126848) and increased in HCC (Fig. 7M–O). However, CIT showed opposite changes in two NASH datasets (GSE17470 and GSE126848) (Fig. 7P, Q). Since NASH can progress to fatty liver fibrosis, we hypothesized that CIT decreased in the early stages of NAFLD and NASH, but its abnormally high expression can mediate the fibrosis of NASH. Analysis of the GSE89632 database containing fibrosis staging data for NASH showed that CIT expression was significantly higher in advanced cirrhosis patients with NASH than in early patients, which is also consistent with the high expression of CIT in HCC (Fig. 7R). However, there was no significant difference in the expression levels of TEAD4 and SOCS2 (Fig. 7F, L). This indicates that the high expression of CIT can mediate the fibrosis progression of NASH, thereby leading to the occurrence and development of HCC, and TEAD4 and SOCS2 may affect HCC through pathways other than liver fibrosis.

### CIT is highly expressed in clinical HCC samples and associated with poor prognosis

**Figure 8:**
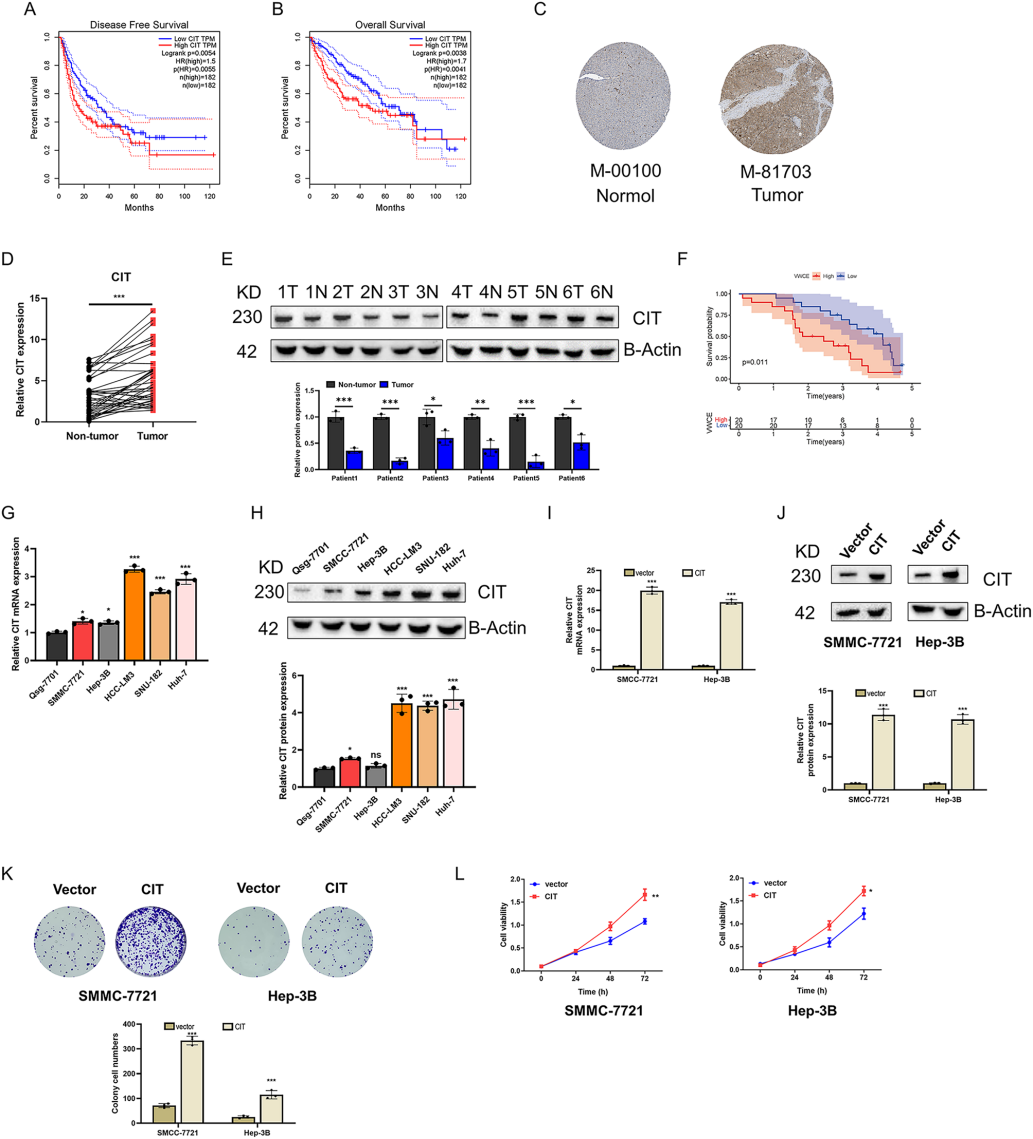
CIT is highly expressed in clinical HCC samples and associated with poor prognosis. (**A**) Survival curves of HCC patients from the TCGA database. (**B**) Disease-free survival curves of HCC patients from the TCGA database. (**C**) IHC results from the HPA database showing the protein expression of CIT in HCC tissues. The mRNA (**D**) and protein (**E**) expression of CIT in 40 HCC and its paracancerous tissues from Guangxi Medical University Cancer Hospital. (**F**) Survival curves of 40 HCC patients from Guangxi Medical University Cancer Hospital grouped based on CIT expression. (**G**, **H**) CIT mRNA and protein levels in HCC cell lines. (**I**, **J**) Establishment of CIT overexpression model. (**K**, **L**) Cell proliferation and CCK8 assays show the proliferation ability of HCC cells.

There is no report of CIT in HCC so far. Survival analysis based on the TCGA database showed that the survival rate and disease-free survival rate of HCC patients with high CIT expression were significantly lower than those of low CIT expression group (Fig. 8A, B). HPA data also showed high protein expression of CIT in HCC tissues (Fig. 8C). Then, we performed qPCR detection on 40 cases of HCC tissues from Guangxi Medical University Cancer Hospital and their paired paracancerous tissues, which showed that CIT was significantly highly expressed in HCC tissues, which was also confirmed by the results of western-blot (Fig. 8D, E). And the high expression of CIT was highly correlated with the poor prognosis of HCC patients (Fig. 8F). Then we found that CIT was highly expressed in all HCC cell lines (Fig. 8G, H). We constructed an overexpression model using the SMCC-7721 and Hep-3B cell lines with the lowest expression (Fig. 8I, J). Cell proliferation assay and CCK8 assay showed that CIT significantly promoted the proliferation ability of HCC cells (Fig. 8K, L).

## Discussion

The ineffectiveness or severe limitations of the current therapeutic targets, which make it difficult to apply them to all patients, provide a problem in the treatment of HCC. Another issue it is difficult to forecast patients' prognoses and make an early diagnosis^14–16^. Therefore, it is a more reliable model to provide patients with early treatment and accurate prediction of prognosis. Using bioinformatics, we screened three genes (TEAD4, SOCS2, and CIT) that may play an important role in the transformation of NAFLD/NASH into HCC, and constructed a risk prognosis model in HCC patients, which is helpful for the early stage of HCC diagnosis and precision therapy.

Our results showed that risk score was an independent prognostic factor for HCC, and HCC patients in the high-risk group had higher T stage and STAGE (Fig. 3A–D). Patients in the high-risk group had worse prognosis, and our model showed good accuracy in four HCC datasets including TCGA, ICGC, GSE116174, and GSE54236 (Fig. 3, 4). In addition, we also constructed a nomogram that is convenient for clinicians to make clinical decisions, and has good prediction accuracy for 1, 3, and 5-year survival prediction (Fig. 4A–D).

There has been evidence that immune abnormalities can lead to the progression of NAFLD or NASH, and even malignant transformation to HCC^17,18^. However, there are still few studies on the malignant transformation of benign diseases mediated by TIME changes, which is very important for the early diagnosis of cancer. Our analysis of immune cell infiltration revealed that genes related to malignant transformation of benign liver lesions are involved in the tumor's immune cell activity and immune microenvironment. The infiltration levels of mast cells, NK cells, and T helper cells in the high-risk group were increased and decreased, which indicated that there was a certain immune escape in the high-risk group. And the cytolytic activity of the high-risk group, IFN class I and II was inhibited, confirming our point (Fig. 6G). By further studying the genes we screened, it is possible to discover new immunotherapy targets.

TEAD4 is a key regulator in the Hippo signaling pathway, controlling the expression of genes involved in cell growth and differentiation^19^. Its upregulation is associated with various cancers, metastasis, and poor prognosis^20,21^. TEAD4 promotes liver cancer cell proliferation, migration, and tumor growth^22^. Activation of Yes-associated protein (YAP) by increased extracellular matrix stiffness leads to direct inhibition of ACADL (Acyl-CoA dehydrogenase long chain) by the YAP/TEAD4 transcription complex. ACADL-dependent mechanosensing pathway is a potential target for treating liver cancer^23^. Notably, TEAD4 shows contrasting expression patterns in NAFLD and liver cancer (Fig. 7A–F), suggesting its regulatory role in the progression from NAFLD to liver cancer. Further research is necessary to explore these findings and their implications on oncogenic signaling pathways like Hippo.

Because of the complexity of the progression from NAFLD/NASH to HCC, it is difficult to pinpoint a precise target. Among the 3 genes related to malignant transformation of benign liver lesions that we screened, only SOCS2 was expressed in NAFLD, NASH, and HCC, indicating that its persistent low expression plays a key role in the progression of NAFLD/NASH to HCC. Suppressors of cytokine signaling (SOCS) are a family of intracellular proteins that act as fundamental physiological regulators of cytokine signaling. A key biological function of SOCS is to mediate cytokine signaling through a negative feedback mechanism. Dysregulation of SOCS has also emerged as an important regulator of inflammatory and oncogenic proliferation pathways^24^. SOCS2 has been shown to protect against chemically induced HCC progression by modulating hepatic inflammation and cell proliferation^25,26^. Another study showed that SOCS2 inhibits inflammation and apoptosis during NASH progression by limiting NF-κB activation in macrophages^27^. These findings confirmed our immune-related analysis (suppression of NK cells in high-risk patients). Since SOCS2 changes the role of immune microenvironment in the development of NAFLD/NASH to HCC, it is very promising to use it as a target to prevent the malignant transformation of NAFLD/NASH in the future, so as to achieve the effect of primary prevention. In addition, due to its important role, its specific drugs are also of great significance in improving the prognosis of patients with advanced HCC.

CIT, a serine/threonine kinase, potentially regulates cancer development by phosphorylating downstream targets involved in apoptosis^28,29^. It exhibits a unique expression pattern and plays a role in hepatocyte cell cycle control^30^. CIT’s role in NAFLD and HCC has not been studied. Our bioinformatics analysis reveals reduced CIT expression in early NAFLD/NASH but significantly elevated expression in late-stage NASH with fibrosis and HCC (Fig. 7M–R). High CIT expression promotes NASH fibrosis and HCC progression (Fig. 8). Clinical samples confirm elevated CIT expression in HCC tissues, correlating with poor prognosis. In vitro experiments demonstrate CIT’s ability to enhance HCC cell proliferation.

Our study provides a reliable risk prognostic model to predict the prognosis of HCC patients. We confirmed the relationship between the model and TIME, and the gene-mediated alteration of immune microenvironment in the model may be closely related to the malignant transformation of NAFLD/NASH. Our research provides support for early prevention and diagnosis of HCC, as well as precise treatment.

### Supplementary Information

Supplementary Information.

## Data Availability

The data that support the findings of this study are available from the corresponding author upon reasonable request. TCGA-LIHC data are publicly available and extracted from https://portal.gdc.cancer.gov/projects/TCGA-LIHC.

## Abbreviations

HCC: Hepatocellular carcinoma
TCGA: The Cancer Genome Atlas
TME: Tumor microenvironment
TIME: Tumor immune microenvironment
