# Supplementary figures and images for: A three-gene signature reveals changes in the tumor immune microenvironment in the progression from NAFLD to HCC
Source: Sci Rep. 2023 Dec 15;13:22295. doi: 10.1038/s41598-023-49358-w (PMC10724126; doi:10.1038/s41598-023-49358-w)

Figure 8E

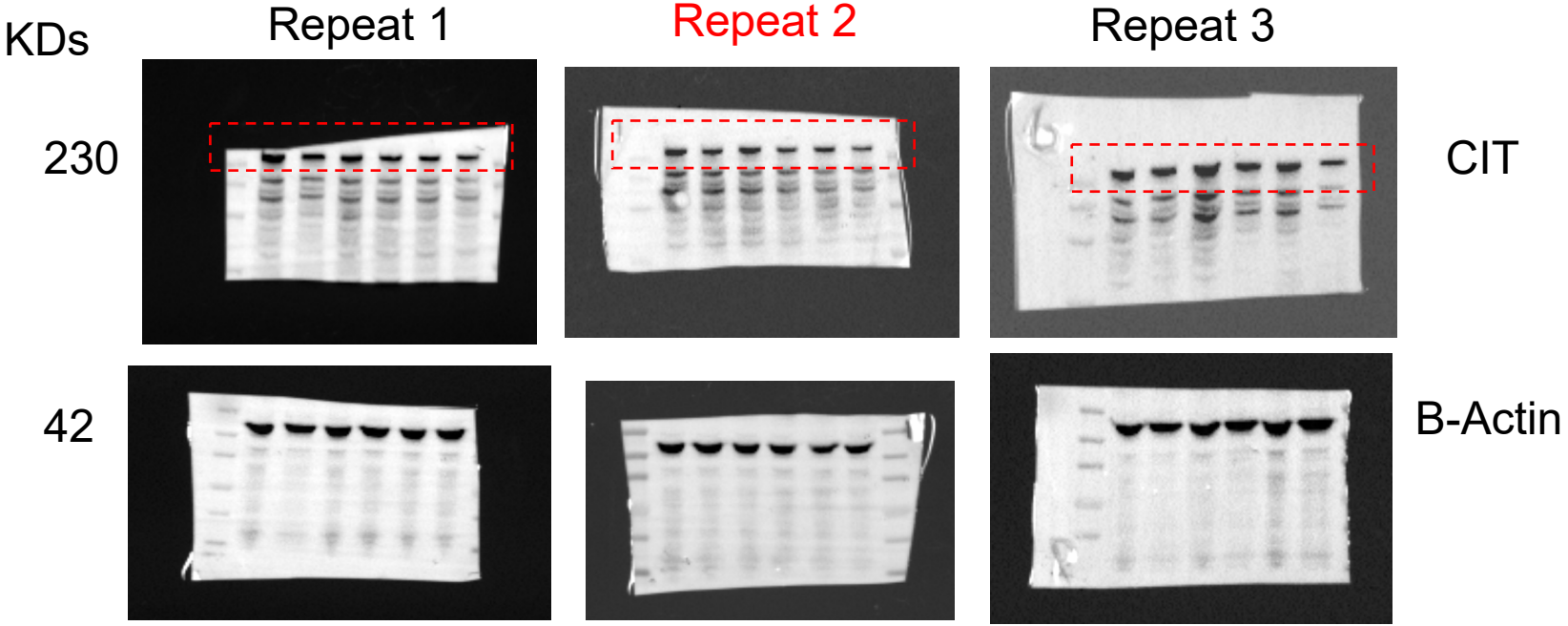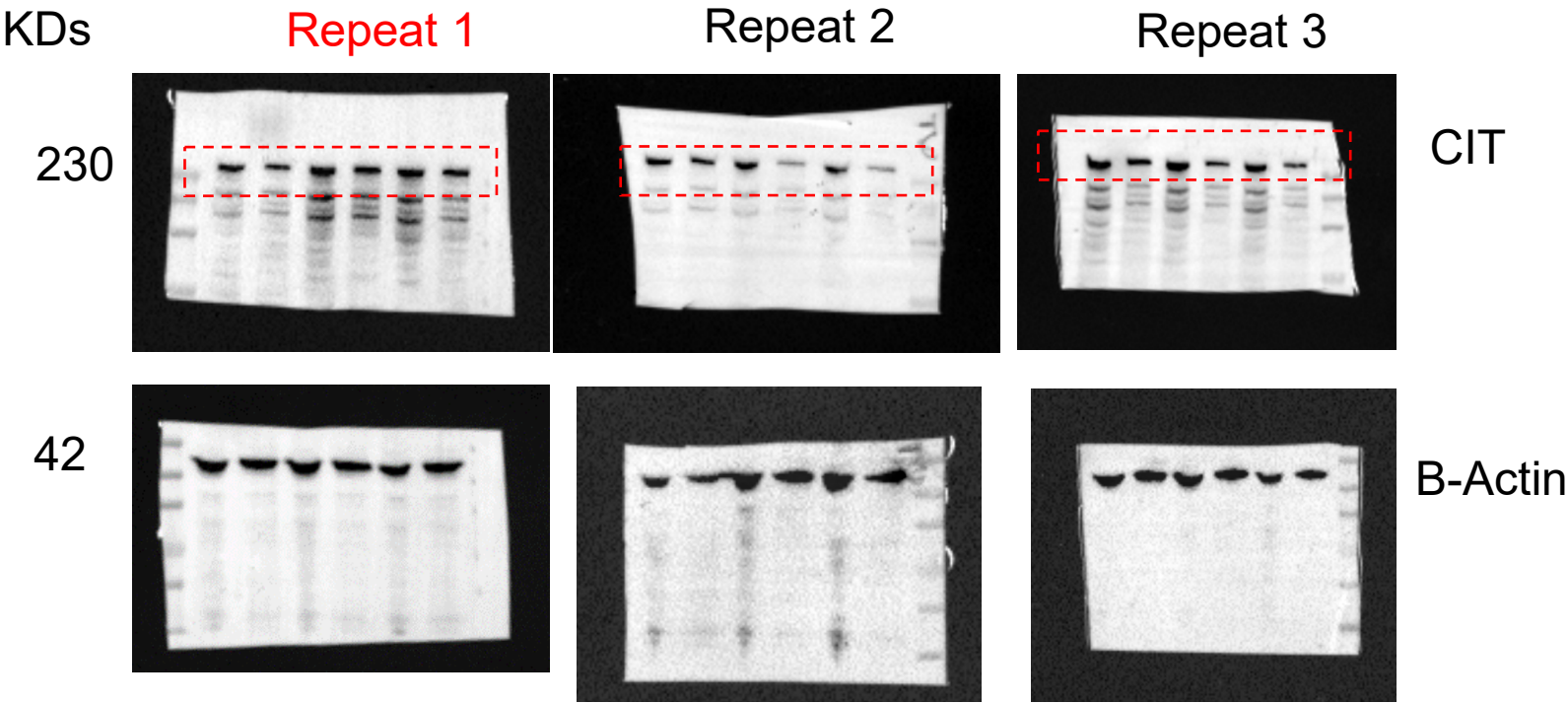

Figure H

KDs

Repeat 1

Repeat 2

Repeat 3

230

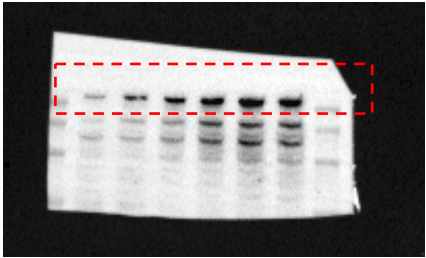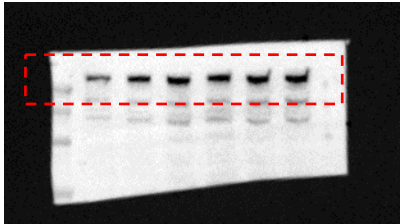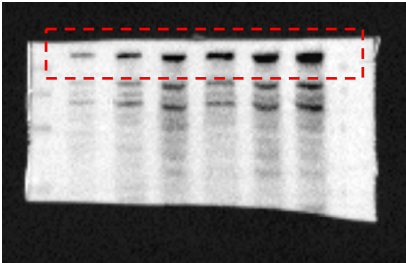

CIT

42

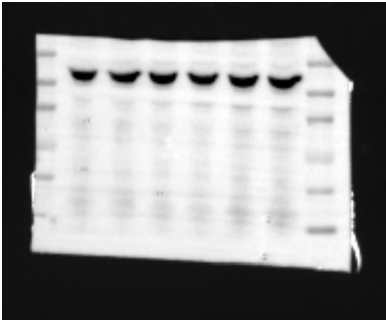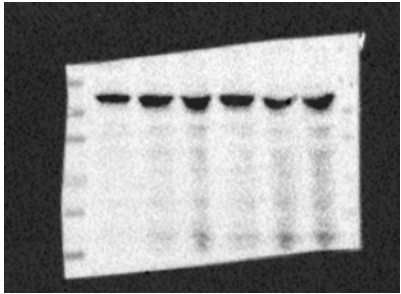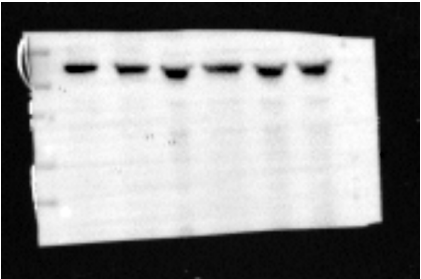

B-Actin

Figure J

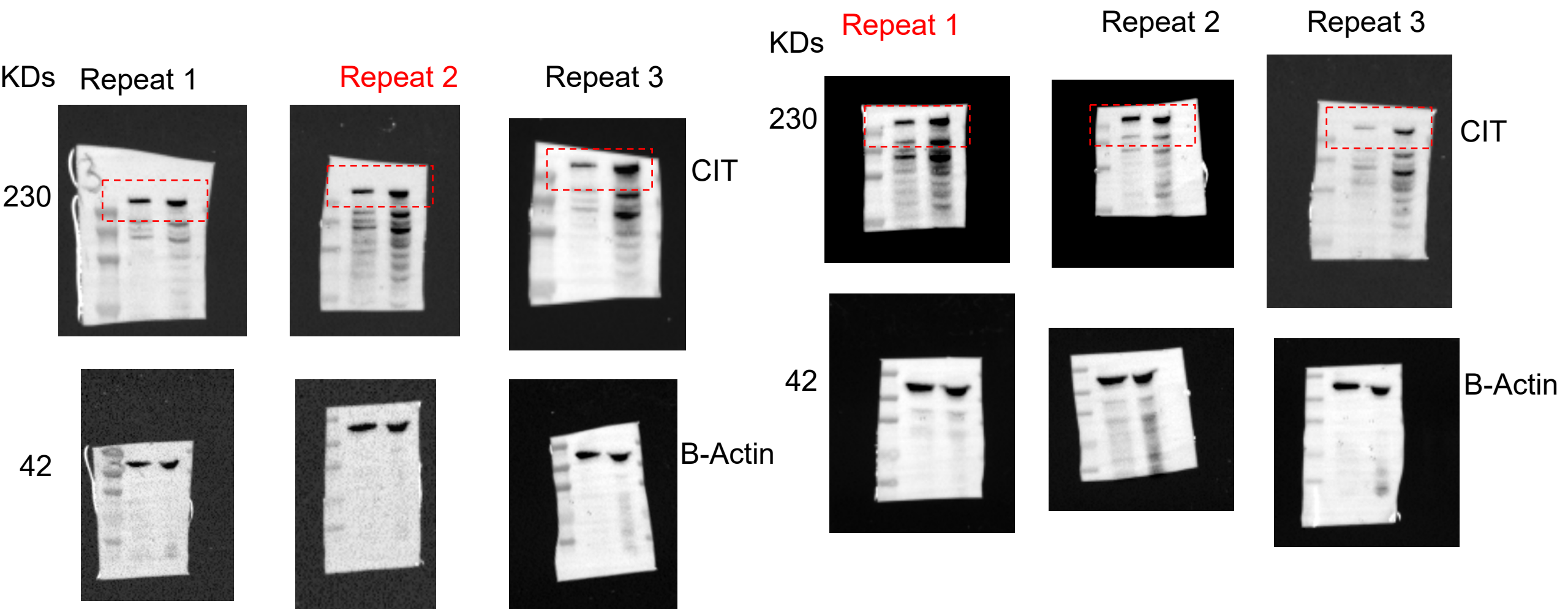

Supplement: Supplementary file 1 — Supplementary Information. [file 41598_2023_49358_MOESM1_ESM.pdf]
